# Volcanic hazard exacerbated by future global warming-driven increase in heavy rainfall

**DOI:** 10.1098/rsos.220275

**Published:** 2022-07-27

**Authors:** Jamie I. Farquharson, Falk Amelung

**Affiliations:** Rosenstiel School of Marine and Atmospheric Science, University of Miami, Miami, FL, USA

**Keywords:** climate change, volcanism, general circulation model, precipitation, geosphere–hydrosphere interaction, climate forcing

## Abstract

Heavy rainfall drives a range of eruptive and non-eruptive volcanic hazards. Over the Holocene, the incidence of many such hazards has increased due to rapid climate change. Here, we show that extreme heavy rainfall is projected to increase with continued global warming throughout the twenty-first century in most subaerial volcanic regions, increasing the potential for rainfall-induced volcanic hazards. This result is based on a comparative analysis of nine general circulation models, and is prevalent across a wide range of spatial scales, from countries and volcanic arcs down to individual volcanic systems. Our results suggest that if global warming continues unchecked, the incidence of primary and secondary rainfall-related volcanic activity—such as dome explosions or flank collapse—will increase at more than 700 volcanoes around the globe. Improved coupling between scientific observations—in particular, of local and regional precipitation—and policy decisions may go some way towards mitigating the increased risk throughout the next 80 years.

## 1. Climate change and volcanism

The role of Earth's subaerial volcanism in driving past climate changes has been substantial [1]—due in large part to the radiative and chemical effects of erupted gases and aerosols [2]—and it is anticipated to drive further variability in the future [3,4]. In turn, variations in climate have also been posited to drive volcanic activity [5–9]. Mechanisms such as the isostatic unloading of the crust due to warming-induced glacial retreat and ice-cap melt [10,11] or crustal stress changes generated by changing sea levels [12] have been proposed to promote volcanic activity over a range of spatio-temporal scales. Over the last 30 ka, changes in climate have driven an increase in massive volcanic collapses, partly in response to increased humidity and rainfall [13]. An uptick in rainfall-driven volcanic hazards has been proposed for many volcanic regions as global climate continues to warm throughout the Anthropocene; in particular, in unglaciated high-relief volcanic environments [7]: an observable rate change in hazardous geological phenomena that may already be under way [14].

Extreme or seasonal rainfall has been identified as a trigger mechanism for primary volcanic activity—discrete eruptions of lava, tephra and gases—at multiple volcanoes. Examples include rainfall-triggered explosions at Mount St Helens (USA), Gunung Merapi (Indonesia) and Las Pilas (Nicaragua) [15–17]. Coupling between extreme rainfall events and dome collapse has also frequently been noted [18–22], with heavy rainfall also being linked to the generation of pyroclastic density currents [21]. More recently, a link between extreme rainfall, pore fluid changes at depth and magma propagation has been proposed at Kīlauea Volcano, USA [23] and Etna, Italy [24]. Rainfall-triggered volcanism is often violently explosive [15], and multiple direct fatalities have been recorded as a result, including at Karkar [25], Guagua Pichincha [26] and Karangetang [27] volcanoes (Papua New Guinea, Ecuador and Indonesia, respectively). Many hazards associated with extreme precipitation events or prolonged rainfall are heightened in volcanic regions: not only do mountainous regions tend to modify and amplify precipitation [28], but they are often mantled by variably consolidated tephra deposits and other easily mobilized debris, and can be associated with large thermal gradients. These gradients drive explosive fuel–coolant interactions [29], and thermal atmospheric forcing due to volcanic thermal anomalies can also increase precipitation above the threshold required to trigger hazards [30]. These factors promote a range of rainfall-related secondary volcanic hazards, including the remobilization of volcanogenic deposits in the form of lahars [31–33] and the instigation of flank mass movement [34–37], a phenomenon that can in turn unload the magma chamber and promote explosive decompression or dyke initiation [38]. Volcanic slopes, typically with low cohesion and narrow grain-size distributions, may be particularly disposed to mass wasting events [37].

**Figure 1.**
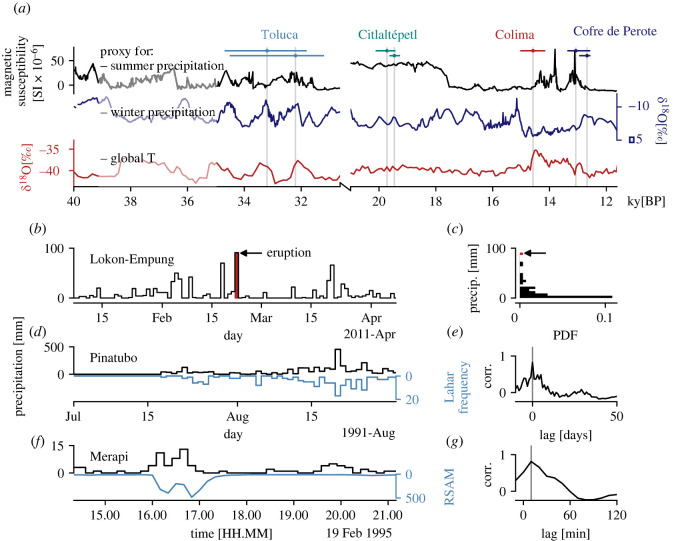
Extreme rainfall as a driver of volcanic hazards. (*a*) Pleistocene volcanic sector collapses of Volcán de Colima, Nevado de Toluca, Citlaltépetl and Cofre de Perote (Mexico), reproduced after Capra *et al*. [39]. Climate proxy data are described in the Material and methods. For each of the seven collapses, horizontal date ranges are indicated, as well as a vertical line highlighting the maximum probability collapse date. Note discontinuous *x*-axis. (*b*) The February 2011 eruption of Lokon-Empung is shown by a vertical line, alongside time series of local precipitation data. (*c*) Log-normal distribution of precipitation data from (*b*), with outlying value (corresponding to date of eruption) indicated. (*d*) Daily precipitation data (black) is plotted against the number of lahars per day (blue) observed at Pinatubo between July and September 1991. (*e*) Result of cross-correlation analysis of Pinatubo data shown in (*d*), shown as correlation coefficient (corr.) between daily precipitation and lahar frequency versus lag. (*f*) Precipitation in ten-minute bins at Merapi volcano, alongside the RSAM value at the same temporal resolution. RSAM maxima reflect peak lahar surges. (*g*) Result of cross-correlation analysis of Merapi data shown in (*f*), shown as correlation coefficient between ten-minute precipitation and RSAM value versus lag. Refer to Material and methods for all data sources.

The timing, distribution and amount of rainfall received by active volcanic systems is influential over a range of timescales. Figure 1*a* indicates catastrophic Pleistocene sector collapses of four volcanoes in Mexico: Volcán de Colima, Nevado de Toluca, Citlaltépetl and Cofre de Perote. In all cases, depositional sequences show evidence of water saturation, hydrothermal alteration and/or water circulation within the pre- and syn-collapse edifice. In light of the lack of systematic concomitant magmatic activity, pluvial conditions have instead been proposed to have triggered these volcanic collapses [39]. Tellingly, each of the events are associated with timeframes characterized by locally high precipitation, typically concurrent with elevated global temperatures. Similar climatic forcing of volcanic collapse has been identified for volcanoes in Europe [40] and South America [41]. Links also exist over shorter timescales: at Lokon-Empung, a triggered volcanic eruption (22 February 2011) coincided with the quarterly rainfall maximum (figure 1*b*,*c*). Figure 1*d* illustrates the intimate correlation between elevated rainfall and lahar generation (i.e. the propagation of potentially devastating pyroclastic slurries) at Mt Pinatubo (Philippines), with a lag of less than 1 day (figure 1*e*). Finally, figure 1*f* shows the hours-to-minutes lahar response (reflected in real-time seismic-amplitude measurement: RSAM) at Merapi. For both Pinatubo and Merapi, cross-correlation analysis reveals that lahar occurrence is related to heavy rainfall with a sub-daily lag (as low as 10 min in the case of the latter: figure 1*e*,*g*). Although figure 1 highlights just a handful of volcanoes, a textual analysis of the Smithsonian's Global Volcanism Program Bulletin Reports—a multi-decadal catalogue of reports of volcanic activity—reveals that extreme or heavy rainfall has been implicated in triggering or exacerbating hazards at at least 174 discrete volcanoes: around 13%, or one in every seven of Earth's subaerial volcanic inventory (see Material and methods).

As the rate of global climate change continues to accelerate, it becomes ever more crucial to develop a comprehensive understanding of the manifold interactions and feedbacks between the atmosphere, cryosphere and solid Earth: complexly interconnected components of the Earth system. Here, we focus on the role of heavy rainfall in volcanic environments, and the evolution of rainfall rates over a multi-decadal timeframe induced by the ongoing rapid changes in global climate. A key problem with identifying volcanic regions at increasing risk has been the inherent uncertainty of climate modelling [7]. While there is broad consensus as to the direction of mean global precipitation change [42,43], global climate models (general circulation models: GCMs)—even when initiated with the same parameters—do not show general concurrence upon the magnitude or spatial distribution of precipitation change, and observations of global mean precipitation changes are at often odds with projected changes [44]. Consistently, however, these models project an increase in the intensity and frequency of heavy precipitation—that is, extreme precipitation events—both on global and regional scales [45]. Fischer *et al*. [46] and Pfahl *et al*. [47] demonstrate that global climate models tend to concur when considering future heavy precipitation. In particular, those authors found that most models tested in their analysis agreed on the sign of change of the diurnal maximum precipitation over time at any given location.

In this contribution, we analyse a suite of numerical global climate models to assess which of Earth's subaerial volcanoes are projected to experience increases or decreases in extreme rainfall, revealing several volcanic systems which we estimate will become more susceptible to rainfall-induced hazards over the next 80 years. In particular, we focus on the forced model response (FMR), the percentage change of heavy precipitation for a given unit of global warming, which serves as a proxy for the likelihood of extreme rainfall events, calculated from nine Coupled Model Intercomparison Project Phase 5 (CMIP5) GCMs (Material and methods).

## 2. Material and methods

### 2.1. Climate proxy and volcanic hazard data

Figure 1*a* is reproduced after Capra *et al*. [39], using magnetic susceptibility data from lake sediment core from Pete-Itzá, Guatemala [48] (interpreted to reflect changes in summer precipitation), speleothem calcite *δ*^18^O data from central New Mexico [49] (interpreted to reflect changes in winter precipitation) and the Greenland Ice Sheet Project 2 *δ*^18^O [50] as a proxy for global temperature. Precipitation data in figure 1*b* from Stasiun Geofisika Winangun (longitude, latitude: 124.83890, 1.44340) were accessed from Indonesia's Meteorology, Climatology and Geophysics Agency (Badan Meteorologi, Klimatologi, dan Geofisika: BMKG) data retrieval portal (https://www.bmkg.go.id/). Daily data of figure 1*d* are from Pierson *et al.* [51]. Merapi rainfall and RSAM data (figure 1*f*) were digitized from Lavigne *et al.* [52].

### 2.2. Textual analysis of bulletin reports

Geolocation data for Earth's subaerial volcanoes are obtained from the Smithsonian's Global Volcanism Program (GVP) databases [53] using the GVP webservices interface. We concentrate on volcanic systems active in the Holocene (discounting volcanoes defined as primarily submarine or subglacial): 1234 volcanoes. The prior association of any particular volcano with rainfall-related volcanic hazard was determined by programmatically querying the catalogue GVP Bulletin Reports for the (case-insensitive) string literals ‘lahar’, ‘heavy rain’, ‘rainfall-triggered’, ‘rainfall-induced’ and ‘extreme rainfall’ (ignoring punctuation and capitalization). The crawled reports were then manually parsed to identify volcanoes with previous evidence for volcanic hazard caused or exacerbated by rainfall, and to remove reports where rainfall was mentioned in non-hazard contexts (for example, reports on the effect of rainfall on monitoring equipment or the volcanic system that do not constitute a clear hazard, geographical background descriptions, or observational and logistical difficulties associated with inclement weather). The remaining catalogue refers specifically to hazards associated with heightened rainfall activity: steam explosions; the instigation of lahars and mudflows; column collapse and pyroclastic density current generation; landslides, rockfalls and other mass wasting events; flooding due to crater lake overflow and triggered primary volcanic activity.

### 2.3. Forced model response

**Figure 2.**
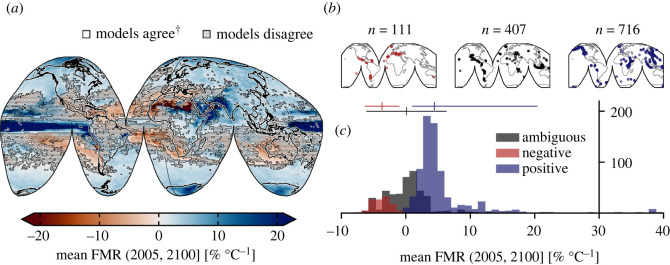
Breakdown of mean forced model response. (*a*) Global mean forced model response (FMR) calculated from all models. Shaded area indicates those regions where fewer than seven of nine models agreed on the sign of change (26.55%). ^†^At least seven of nine models agree on the sign of change. (*b*) Subaerial volcano geolocations separated according to whether models agree on a decrease in heavy precipitation with increased warming (red: ‘negative’; *n* = 111); the precipitation response is ambiguous due to lack of model agreement (black: ‘ambiguous’; *n* = 407); models agree on an increase in heavy precipitation with increased warming (blue: ‘positive’; *n* = 716). *n* indicates the number of discrete Holocene-active volcanic systems in each category. (*c*) Histogram of mean FMR for each group of volcanoes (as in (*b*)). Mean and two standard deviation range are indicated by the vertical and horizontal lines, respectively (Material and methods).

**Figure 3.**
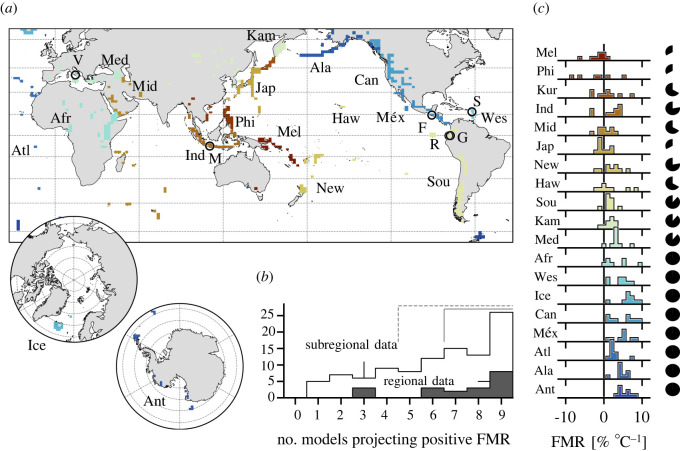
Regional and sub regional spatial averages. (*a*) Map indicating the non-contiguous spatial extent over which regional data are averaged. Circle markers indicate individual volcanoes shown in figure 4. V, Vesuvius; M, Merapi; F, Fuego; R, Reventador; G, Guagua Pichincha; S, Soufrière Hills Volcano. [Inset] polar regions. Regions are represented by discrete coloured rectilinear polygons. Ant, Antarctica; Atl, Atlantic Ocean; Sou, South America; Ala, Alaska; Kur, Kuril Islands; Ind, Indonesia; Mid, Middle East and Indian Ocean; Phi, Philippines and SE Asia; Méx, México and Central America; Jap, Japan, Taiwan and Marianas; Kam, Kamchatka and Mainland Asia; Med, Mediterranean and Western Asia; New, New Zealand to Fiji; Haw, Hawai`i and Pacific Ocean; Ice, Iceland and Arctic Ocean; Afr, Africa and Red Sea; Wes, West Indies; Mel, Melanesia and Australia; Can, Canada and Western USA. (*b*) Bar chart of the number of regions and subregions where *x* number of models project a spatially averaged forced model response (FMR) > 0 (i.e. a concomitant increase in heavy precipitation and global mean temperature). Dashed bracket indicates the majority of models, solid bracket indicates seven or more out of nine models. (*c*) Inter-model distributions of calculated FMR for each region. Marginal pie charts indicate the proportion of models that project a positive FMR per region (out of maximum of nine).

**Figure 4.**
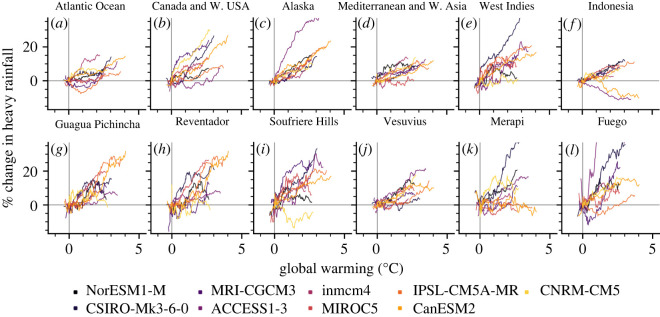
Forced model responses at different spatial scales. (*a–f*) Per cent change in modelled heavy rainfall per degree of global warming. Data are shown as a 30-year rolling mean, normalized to January 2021. Data are areal averages (figure 3 for areal extent of each region). (*g–l*) As (*a–f*), for individual volcanic systems. Data correspond to the bounding pixel for each model (see Material and methods). Volcano locations are shown in figure 3.

Ensemble climate projection experiment data were obtained from the CMIP5. CMIP5 comprises a set of coordinated climate model experiments, performed by several independent modelling groups using more than 50 discrete Earth System models, with the goal of providing a multi-model assessment of simulated climate change (and variability thereof) over timescales from decades to centuries. For a more comprehensive background to CMIP5, the reader is referred to Taylor *et al.* [54]. Here, we use data from nine separate models (an ‘ensemble of opportunity’ [55]), listed in electronic supplementary material, table S1, each of which follow the Representative Concentration Pathway (RCP) 8.5 scenario (a ‘high emissions’ scenario). The total period covered by the selected data is from 2005 or 2006 to 2100. For comparability, we use models from ensemble r1i1p1 only (i.e. the initial conditions and the constitutive model physics are the same, and differences in simulations reflect internal inter-model variability), at a monthly frequency. For each model and each year over the modelled period, we calculate the mean global temperature 〈*T*〉 timeseries and the maximum monthly rainfall value *RXm* for each grid cell. The FMR is calculated as the slope of a linear regression of *RXm* versus 〈*T*〉 normalized to 1 January 2006 (figure 2, electronic supplementary material, figure S1) or 1 January 2021 (figures 3 and 4). The resulting two-dimensional array *A_k_*, where *k* is the number of the model, has dimensions dependent on the initial spatial resolution of the model experiments (electronic supplementary material, table S1). For each model *k*, the value of each cell at latitude *i* and longitude *j* is binarized such that *B_ijk_* = *H*(*A_ijk_*) where *H*(*x*) is the Heaviside function and the Boolean units 0 and 1 thus denote negative and positive FMRs, respectively. To determine areas where the majority of models agree on the sign of heavy precipitation change, we resample the binary arrays onto a common 180 × 360 grid (i.e. approx. 1 × 1°) using a nearest-neighbour approach, then sum them such that $C=\sum_{k=1}^{n=N}B_{k}$. Agreement in the sign of normalized *RXm* across at least seven of nine models is represented by |*C_ij_* − (9/2)| > 2, where *C_ij_* ∈ [0, 9]. This criterion (7/9 models or 78% model agreement) is comparable to the threshold imposed by previous studies [46,47]. Calculated FMRs from the individual CMIP5 GCMs are shown in electronic supplementary material, figure S1.

### 2.4. Distribution statistics and other calculations

Additional analyses were performed on an *ad hoc* basis for individual systems or sets; for completeness, these methods are described here. Where appropriate, maxima and mean volcano flank slope values were calculated using the database compiled by Grosse *et al.* [56]. Uniformity was tested for using the chi-squared (*χ*^2^) method. Statistical significance was ascribed where the cumulative distribution function of the chi-squared statistic CDF(*χ*^2^) was less than 0.01. Descriptive statistics of volcano FMR distributions (figure 2*c*) were calculated assuming a normal distribution (negative and ambiguous) and a log-normal distribution (positive). Cross-correlation analysis of Pinatubo and Merapi lahar data was performed by treating rainfall and lahar data as one-dimensional sequences. Figure 1*e*,*g* shows the correlation coefficient for each lag value, in days (Pinatubo) or minutes (Merapi). Correlation maxima are 0 days and 10 min, respectively, indicating a relatively short lag between heavy rainfall and lahar occurrence at both sites. All analyses were performed in Python.

## 3. Results and discussion

### 3.1. Climate models agree on the direction of heavy precipitation change with global warming

Calculated FMRs from the individual CMIP5 GCMs are shown in electronic supplementary material, figure S1, presented in % °C^−1^ as the gradient of a regression between monthly heavy precipitation change *RXm* and global mean temperature 〈*T*〉. There is qualitative agreement in many areas across models: less extreme rainfall is forecast by most models for the majority of Australia, parts of Saharan and southern Africa and Central America, for example, whereas large portions of North America, Eurasia, East Africa and the Polar regions are projected to experience an increase in extreme precipitation with continued global warming. This is emphasized by mean response of all models resized onto a common grid (figure 2*a*). The areas where fewer than seven of nine models agree on the sign of FMR are shaded. The area over which at least seven of nine models concur accounts for 73.45% of the globe, in line with previous multi-model studies [46,47], despite the fact that the cited studies examine models at a daily resolution over longer timescales (including historical simulations) and analyse more models (15 and 22, respectively). As well as the proportion of model agreement, we highlight that the areas of agreement are qualitatively similar to those studies. In a volcanic context, regions where extreme rainfall is projected to increase account for large portions of each of the continental volcanic arcs (the Cascades, the Alaskan Peninsula and Aleutian Range, Kamchatka and Northern and Central Andes), parts of the Mediterranean and East African Rift system, and throughout the Sunda, Philippine, Ryuku, Japan, Kuril, Aleutian and West Indies island arcs. Smaller subtropical island arcs, including the Bismarck Archipelago are also encompassed. On the other hand, models tend to agree that extreme rainfall will decrease in parts of the Southern Andean Volcanic Zone and Rangitāhua (the Kermadec Islands), for example.

Of the 1234 Holocene-active subaerial volcanic systems included in the initial dataset, 716 (58%) are situated in regions with a positive FMR (i.e. regions that are forecast to experience more extreme rainfall over the next 80 years) across the majority of GCMs (figure 2*b*). Two hundred and forty-four of these (19% of the initial dataset) have a mean (averaged over all models) FMR ≥ 5% °C^−1^. Nineteen volcanoes (1.5%) exhibit a mean FMR ≥ 20% °C^−1^, all of which are located in the Galápagos, the East African Rift and Papua New Guinea, between 3.125 °S and 25.000 °N. Highlighted in figure 2*b*, only 111 volcanoes (9%) are located in regions anticipated to experience less extreme rainfall, with the remaining 407 (33%) being associated with an ambiguous FMR (where fewer than seven of the nine models agreed with the sign of heavy precipitation change). We note that the proportion of volcanic systems associated with positive or negative FMR changes negligibly if the grid size is arbitrarily reduced. The aggregate FMR distribution of each of the models is approximately symmetrical around a median of 3.2% °C^−1^, indicating that the majority of the globe is projected to experience an increase in extreme rainfall. When we consider only those grid cells containing active volcanic systems (figure 2*c*), we observe a log-normal distribution of volcanoes with positive FMR, with a mean value of approximately 4.5% °C^−1^ and a long tail on the positive side: the substantive majority of Earth's subaerial volcanic systems will be subject to more extreme rainfall with every increment of global warming over the remainder of the twenty-first century.

### 3.2. Models project an increase in heavy precipitation for most or all volcanic regions

The GVP subdivides Earth's volcanoes into 19 discrete regions, which are further subdivided into 101 subregions. Extracting areal averages of these volcanic regions (those grid cells containing at least one Holocene-active volcano: discrete coloured rectilinear polygons in figure 3*a*), we calculate the linear regression-based gradient of change in heavy precipitation versus global warming. A summary of the results is given in table 1.

**Table 1.** Model analysis results. Abbreviation corresponds to the three-letter code on figure 3. *n* is the number of historically active volcanoes within the region. Mean and median FMR values are given, along with standard deviation from the mean. ‘min’ and ‘max’ refer to the minimum and maximum calculated values of FMR for each region. ‘# +ve’ refers to the number of models (out of nine) that yield a positive FMR value (figure 3*c*).

region			FMR					
abbr.	name	*n*	mean	s.d.	median	min	max	# +ve
Mel	Melanesia and Australia	66	−3.04	4.97	−0.98	−15.87	1.16	3
Phi	Philippines and SE Asia	47	−3.02	7.58	−2.54	−13.41	10.84	3
Kur	Kuril Islands	41	0.97	3.35	0.79	−3.57	7.78	6
Ind	Indonesia	125	1.68	2.72	2.92	−3.39	4.23	7
Mid	Middle East and Indian Ocean	41	0.49	1.93	0.50	−3.02	3.63	6
Jap	Japan, Taiwan and Marianas	105	−0.08	1.28	−0.56	−2.28	2.24	3
New	New Zealand to Fiji	30	1.93	2.36	1.60	−1.73	6.18	7
Haw	Hawai`i and Pacific Ocean	6	4.56	8.18	1.18	−1.59	25.93	6
Sou	South America	182	1.67	1.45	1.48	−0.90	4.63	8
Kam	Kamchatka and Mainland Asia	85	1.45	1.12	1.63	−0.54	3.03	8
Med	Mediterranean and Western Asia	38	3.09	1.87	2.90	−0.14	7.38	8
Afr	Africa and Red Sea	119	6.24	5.53	5.40	0.44	16.43	9
Wes	West Indies	15	5.01	2.85	5.12	0.61	10.94	9
Ice	Iceland and Arctic Ocean	27	6.55	2.36	6.81	0.69	9.48	9
Can	Canada and Western USA	64	5.16	2.92	6.06	0.87	9.21	9
Méx	México and Central America	109	5.72	3.11	5.58	0.99	12.02	9
Atl	Atlantic Ocean	23	2.78	1.72	2.27	1.23	7.44	9
Ala	Alaska	86	5.25	2.70	4.61	1.25	11.86	9
Ant	Antarctica	25	5.38	1.37	4.92	3.57	8.05	9

For each region, figure 3*b* indicates the distribution of models (out of a maximum of nine) that project a positive FMR: a concomitant increase in heavy rainfall with global warming. For the vast majority of volcanic regions (16/19: 84%), most models project positive FMR. Of these, 13 (64%) exhibit agreement across at least seven models, and for eight regions (Antarctica; Atlantic Ocean; Alaska; Africa and Red Sea; México and Central America; Iceland and Arctic Ocean; West Indies; Canada and Western USA) all models forecast a positive FMR (42% of all regions). There are zero volcanic regions for which at least seven of nine models project a negative FMR (cf. inset pie charts of figure 3*c*). This trend is echoed at the subregional scale (figure 3*b*): the majority of models forecast positive FMR for 74 of 101 subregions (73%), and of these, 54 (53%) exhibit agreement between at least seven models. At both the region and subregion scale, the observed distributions are statistically non-uniform, characterized by CDF(*χ*^2^) ≪ 0.01. Figure 3*c* shows the distribution of calculated gradients across models for each region. Note that majority-positive FMR distributions (e.g. Antarctica, Alaska, Atlantic Ocean, Mediterranean and Western Asia, Kamchatka and Mainland Asia: figure 3*c*) tend to be relatively tightly clustered, whereas for those regions where FMR is predominantly negative or ambiguous (e.g. Philippines, Kuril Islands, Hawai`i and Pacific Ocean: figure 3*c*), the distribution tends to be broader. The proportion of models exhibiting a positive FMR is indicated for each region by the marginal pie charts. We note that for eight regions, all models project a positive regional FMR (see also table 1). Together, this emphasizes the fact that when we observe reasonable inter-model concurrence in any given region, the result is usually that heavy rainfall is set to increase over the next 80 years.

Illustrative examples of regionally averaged climate projections are given in figure 4*a–f*, highlighted here due to the demonstrable risk of rainfall-induced hazard therein (data for all regions and subregions are provided in electronic supplementary material, figure S6). The Atlantic ocean volcanic region (figures 3*a* and 4*a*) largely comprises island volcanoes characterized by a history of catastrophic collapse—including Tristan de Cunha, El Hierro and Tenerife—a potential tsunamigenic hazard facilitated by wet climates [57]. The Canada and Western USA volcanic region (figures 3*a* and 4*b*) is predominantly composed of stratovolcanoes in the Cascade Range. The incidence of sector collapse at several Cascadian volcanoes (including Mount St Helens, Mt Adams and Mt Baker) has been proposed to be triggered or exacerbated by historical climate change, including the attendant increase in humidity and rainfall [13]. Numerous volcanoes in the Cascade Range currently present a significant lahar threat to major population centres [58], with several exhibiting flank segments in excess of 20° slope pitch [56]. Notably, direct evidence of rainfall-triggered explosive activity has been reported for Mount St Helens [15]. The Alaska region (figures 3*a* and 4*c*)—including the Alaskan Peninsula, Aleutian Range and Aleutian island arc—hosts volcanoes with the highest mean and partial flank inclines (in excess of 30° and 40°, respectively [56]. Holocene climate change has already been shown to have driven geologically recent volcanic sector collapse in parts of the Mediterranean and Western Asia region (figures 3*a* and 4*d*) [40], with these areas highlighted as becoming increasingly hazard-prone in the future [14]. The West Indies region (figures 3*a* and 4*e*) has similarly been highlighted [14], and hosts frequently active volcanoes such as Soufrière Hills where primary volcanic activity is observably triggered by heavy rainfall [18,21]. Finally, Indonesia (figures 3*a* and 4*f*)—the world's most volcanically active country and a volcanic region unto itself—is home to multiple volcanoes where explosive behaviour has been triggered by heavy rainfall. Notable examples are provided by excerpts from Smithsonian Institution's GVP Bulletin Reports:

This first Bulletin report discussing Egon describes the sudden appearance of volcanic activity there in January 2004. Heavy rains fell over Egon and its surrounding area on 28 January … followed at 1700 by an explosion and a black ash cloud rising *∼*750 m above the summit [59];

A sudden eruption at Karangetang on 6 August 2010 occurred without warning and caused considerable damage … four people were confirmed dead and five were injured … [An official noted] that the volcano erupted just after midnight when water from heavy rains had penetrated the volcano's hot lava dome, causing the explosion. [27];

The phreatic eruption [of Lokon-Empung] was triggered by extensive rainfall; specifically, 602 mm of rain fell during January 2002 compared to 193 mm during December 2001. This excessive rainfall was thought to cause instability of the edifice. [60];

[O]n 22 February 2011, a phreatic eruption [of Lokon-Empung] … was possibly triggered by high rainfall [61].

Clearly, each of these volcanic regions appears particularly hazard-prone in terms of heavy rainfall-driven phenomena. Just as clearly, heavy rainfall is projected to increase in these regions by most or all climate models, thus heightening an already considerable threat to life, property and infrastructure in the coming decades.

### 3.3. Climate change-induced hazards at individual volcanoes

Figure 4*g–l* presents the FMRs at the scale of individual volcanic systems: Guagua Pichincha and Reventador, Ecuador; Soufrière Hills Volcano, Montserrat; Vesuvius, Italy; Gunung Merapi, Indonesia and Fuego (Chi Q‘aq’), Guatemala. These six volcanoes are chosen due to particularities of their eruptive histories, each of which illustrates the potential for increased hazard in the face of increased heavy precipitation. At Guagua Pichincha (figures 3*a* and 4*g*), cycles of explosivity have been anecdotally attributed to the timing of the rainy season [26]. A violent explosive eruption in 1993, triggered by ‘abnormally high’ rainfall, resulted in the death of two volcanologists. Reventador (figures 3*a* and 4*h*), one of the most active volcanoes in Ecuador, is situated in a cloud-forest region already characterized by extremely heavy rainfall. Combined with its steep slopes [56], these factors contribute to the generation of frequent, often destructive, lahars. An analysis of Reventador's historical eruption catalogue indicates a tendency towards erupting between December and May, when the volcano receives the majority of its annual rainfall. Soufrière Hills Volcano (figures 3*a* and 4*i*) is characterized by sensitivity to heavy rainfall: not only does lahar probability scale directly with rainfall intensity [62], but triggered primary volcanic activity has been reported frequently [18,20,21]. At Vesuvius (figures 3*a* and 4*j*), textural, geochemical and anecdotal evidence of external water—possibly of meteoric origin—exists for several previous large eruptions [63,64]. As with Reventador, we note a tendency for large historic eruptions to occur between July and December. In 1998, a protracted period of extreme rainfall mobilized pyroclastic debris from Vesuvius and the Campi Flegrei systems and generated devastating debris flows, resulting in 160 fatalities with many more injured or displaced [65]. A statistical correlation between intense rainfall and explosive dome collapse has been reported at Gunung Merapi [16] (figures 3*a* and 4*k*). The risk of lahars at Merapi—invariably driven by rainfall [52]—is substantial, with lahar deposits covering an area of almost 300 km^2^ in the region. Rainfall-triggered lahars at Merapi have been responsible for many deaths and the destruction of thousands of homes. The 2010–2011 rainy season at Merapi was not only associated with a cumulative rainfall amount more than 5 m greater than any year in the preceding decade (fostered by a strong La Niña period), but also a substantially higher lahar frequency than following previous eruptive events (as many as 59 in a single month [66]). Finally, at Fuego (figures 3*a* and 4*l*), heavy rainfall has been related to a host of eruptive and non-eruptive hazards, triggering plume emissions, seismic activity and tilt changes [67], as well being directly related to frequently triggered lahars. With climate models almost exclusively projecting an increase in heavy precipitation with continued warming for each of these systems, it is highly probable that the already substantial risk to people, property and infrastructure at these systems will be further amplified in the coming decades.

### 3.4. Lower representative concentration pathway scenarios

RCP 8.5 is a greenhouse gas concentration trajectory associated with high and continuing emissions. For completeness, the analyses outlined above (Material and methods) were also repeated using lower concentration pathways: RCP 4.5 and RCP 2.6. Results from individual and compiled model analysis for RCP 4.5 are provided in electronic supplementary material, figures S2 and S3, and those from individual and compiled model analysis for RCP 2.6 are provided in electronic supplementary material, figures S4 and S5. RCP 4.5 is an intermediate scenario whereby emissions peak around 2040 before declining in the latter half of the century. This additional complexity results in globally lower agreement between models: we retrieve an agreement in the sign of FMR across 60.46% of the globe (compared with 73.45% for RCP 8.5). In turn, this means that it is not possible to obtain a robust estimate of the sign of FMR (i.e. agreement across seven or more models) for most (52%) of the volcano catalogue used. However, it is evident that the proportion of volcanoes located in areas forecast to experience more extreme rainfall using the RCP 4.5 scenario remains significantly higher than volcanoes expected to experience less extreme rainfall: 506 volcanoes (42% of the subaerial catalogue) versus 81 (7%), respectively—a factor of six greater. RCP 2.6 is a ‘very stringent’ pathway, which requires that global carbon dioxide emissions decrease to zero by 2100. Only seven of the climate models examined here (electronic supplementary material, table S1) are available for RCP 2.6; accordingly, we define the threshold for inter-model agreement as 5/7 (i.e. 71.43%: note that this is lower than that for RCP 8.5 and RCP 4.5, and that the results are not directly comparable). In this case, only 46.20% of the globe meets this criterion, with the result that 64% of the volcano inventory is not associated with a clear positive or negative FMR. Nevertheless, 34% of the subaerial volcano catalogue is associated with increasing extreme rainfall under this scenario, compared with only 2% associated with less: a factor of 16 higher. In addition, there is broad qualitative agreement in the geographical distribution of positive and negative FMR, both between CMIP5 models for each RCP (electronic supplementary material, figures S1, S2 and S4) and between the RCP 8.5, RCP 4.5 and RCP 2.6 datasets (figure 2, electronic supplementary material, figures S3 and S5).

These additional analyses highlight that increasingly extreme rainfall in volcanic environments should be anticipated as the norm rather than the exception over the coming century—irrespective of the particular climate future modelled. The increased inter-model discrepancies coincident with decreasing RCP scenarios illustrate the inherent complexity in determining warming-driven geologic hazards; in particular, the potential for large multi-model ranges in projected outcomes is an inherent challenge in the intercomparison of an ad hoc ensemble of models [55]. Nevertheless, the proportion of volcanic systems projected to experience more extreme rainfall relative to the 2006 baseline is consistently greater than those for which extreme rainfall is projected to decrease, even under ambitious climate change mitigation strategies.

## 4. Conclusion and perspectives

In summary, we find that the majority of Holocene-active subaerial volcanic systems (716 volcanoes: 58%) are confidently projected to experience more extreme rainfall as global temperatures continue to rise. Moreover, in some volcanic areas, heavy precipitation is projected to increase by as much as 46% relative to the 2006 value for every degree of warming experienced over the next 80 years, based on a high-emissions climate future. For another 33% of volcanoes globally (in particular at mid-latitudes), there is not sufficient inter-model consensus to confidently estimate whether rainfall will become more or less extreme in the future. Broadly, these results hold across multiple emissions scenarios, and ultimately point to significant attendant implications for rainfall-related hazards at most of Earth's subaerial volcanic systems.

Multi-decadal catalogues of reports of volcanic activity reveal that rainfall has historically triggered, facilitated or worsened primary volcanic activity or secondary hazards at over 170 subaerial volcanoes; a strong reminder that the influence of the hydrological cycle in volcanic systems can be substantial (see also figure 1). This link emphasizes the importance of considering rainfall in the development of hazard mitigation strategies [21,62,68], and also underscores the importance of developing novel instrumental monitoring systems [69,70]. The incorporation of meteorological data into volcano monitoring systems has seen some limited adoption [71]; nevertheless, meteorological data is far from being a standard monitoring tool. Moreover, these results highlight the need for more interdisciplinary volcanological training, as proposed by Fink & Ajibade [72]: compound disasters necessitate an increased range of expertise to effectively mitigate and manage. A number of updated climate models—i.e. the Coupled Model Intercomparison Project phase 6 (CMIP6) generation of GCMs—yield even greater projected warming than the CMIP5 models, although observationally constrained CMIP6 results align more readily with their CMIP5 counterparts [73]. The latter point notwithstanding, high-end global warming scenarios cannot be discounted; rather, they emphasize the need for urgent adaption strategies [74].

While emphasis has been placed previously on the effect of climate change on tropical volcanoes [75], we note that an increase in heavy precipitation is projected to occur with warming in many polar and temperate volcanic regions as well, including the Aleutian Arc, Western USA and Canada, and Antarctica and the South Sandwich Islands, as well as arid regions such as north Africa (electronic supplementary material, figure S6). In resolving cross-model agreement at regional and local scales relevant for volcanic hazard, we demonstrate an explicit, geographically widespread link between global warming scenarios and the potential for increased volcanic hazard. We have not accounted for the influence of global warming on the dynamics of eruption plumes [76], nor for the proposed orographic feedback between heated volcanic summits and precipitation [77] which may serve to further exacerbate the influence of rainfall in volcanic regions. Moreover, it is inevitable that the volcanic response to increasingly extreme rainfall patterns will be strongly dependent on tectonic setting as a key determinant of the nature of hazard exhibited at any given volcano: a level of complexity that is not addressed here in detail. While previous studies have linked rainfall to variations in eruptivity at basaltic shield volcanoes [23,78,79], the majority of quantitative evidence of rainfall-induced volcanic hazard comes from intermediate, dome- and lahar-forming systems such as Soufrière Hills Volcano [18,80,81], Gunung Merapi [16,66] or Unzendake [82]. Further targeted research of the role of extreme rainfall in other settings (e.g. continental rift zones) may provide invaluable context as to the sensitivity of individual systems or volcanic regions. We highlight that broader feedback mechanisms have also been proposed, including climate change-induced perturbations in crustal stress caused by ice sheet and glacier wastage [14], changes to axial and spin-rate of the Earth and realignment of the geoid [8,83], and rising sea levels [84,85], each of which have the potential to trigger subaerial volcanism.

In focusing on extreme climate indices here, we do not quantify the absolute amount of precipitation within the hydrogeological system at a given time. There is, therefore, additional complexity involved in mechanisms which involve a threshold cumulative amount of rainfall to enter the system, or rely on pre-existing system criticality. Quantifying any climate change-induced increase in volcanic activity is non-trivial, and the geospheric response to global warming and an increase in heavy precipitation will certainly be geographically variable [75]. Nevertheless, we may look to Earth system responses to previous long- and short-term changes in climate (e.g. figure 1*a*) to provide some insight into the future [86] where a committed global warming of 1.5–2°C by 2100 appears inevitable [87].

## Data Availability

Data and relevant code for this research work are stored in GitHub: https://github.com/jifarquharson/Farquharson_Amelung_2022_RSOS/ and have been archived within the Zenodo repository: https://doi.org/10.5281/zenodo.6803593 [88]. This includes links to relevant open access repositories from which data were accessed. Model output data have been obtained through Earth System Grid Federation servers, in particular the node hosted by the Lawrence Livermore National Laboratory (https://esgf-node.llnl.gov/search/cmip5/). Data generated in the present study are available at the following repository: https://doi.org/10.5281/zenodo.6803593. Additional datasets supporting this article have been uploaded as part of the electronic supplementary material [89].
